# Quantifying International Travel Flows Using Flickr

**DOI:** 10.1371/journal.pone.0128470

**Published:** 2015-07-06

**Authors:** Daniele Barchiesi, Helen Susannah Moat, Christian Alis, Steven Bishop, Tobias Preis

**Affiliations:** 1 Department of Mathematics, University College London, London, United Kingdom; 2 Warwick Business School, University of Warwick, Coventry, United Kingdom; University of Maribor, SLOVENIA

## Abstract

Online social media platforms are opening up new opportunities to analyse human behaviour on an unprecedented scale. In some cases, the fast, cheap measurements of human behaviour gained from these platforms may offer an alternative to gathering such measurements using traditional, time consuming and expensive surveys. Here, we use geotagged photographs uploaded to the photo-sharing website *Flickr* to quantify international travel flows, by extracting the location of users and inferring trajectories to track their movement across time. We find that *Flickr* based estimates of the number of visitors to the United Kingdom significantly correlate with the official estimates released by the UK Office for National Statistics, for 28 countries for which official estimates are calculated. Our findings underline the potential for indicators of key aspects of human behaviour, such as mobility, to be generated from data attached to the vast volumes of photographs posted online.

## Introduction

The vast amount of data produced by people’s interaction with large technological systems, such as the Internet, has recently begun to enable analysis of collective behaviour on an unprecedented scale. Recent studies in the emerging discipline of computational social science have demonstrated that new online data sources can be mined to unveil previously undocumented human behavioural patterns, reveal networks of social contacts, and forecast collective phenomena emerging from complex socioeconomic systems [1–6].

The value of data captured in large-scale records from the Internet and mobile phone networks has been demonstrated in the study of language and its evolution [7–10], in understanding how social links form [11], in monitoring the spread of diseases [12, 13], and in deriving new socioeconomic indicators [14]. Evidence has recently been presented that datasets generated through human online interactions may be of value in gaining insights into current and future human behaviour across a range of domains, including financial markets [15–17], political elections [18, 19] and consumer behaviour in general [20–23].

Estimates of the number of people visiting a country and their countries of origin are of vital importance to commercial and governmental stakeholders. However, such estimates are currently gathered through time consuming surveys that require substantial human resources. In contrast, data garnered from usage of online services tend to be cheap and available with minimal delay. Here, we use data from the photo-sharing website *Flickr* to quantify international travel flows, and compare our results to official statistics produced by the UK Office for National Statistics. We provide evidence that monitoring the trajectories of *Flickr* users, by analysing geographic information attached to the photos they upload, can provide timely estimates of the movement of humans between countries, complementing more traditional survey-based methods for inferring human mobility patterns.

In previous studies into human mobility, data on the location of mobile phone users has been exploited to reveal universal patterns in the way in which humans travel [24], and to provide quantitative evidence that users who are closer in space are more likely to form social ties [25]. Further mobility research has begun to draw on online data [26], for example demonstrating that the choice of pages which *Wikipedia* editors modify can reveal their location [27].

Increasingly however, communication via online social networks is not only based on text based messages, but also involves uploads of large amounts of photographic and video based media. Previous research has analysed the tags and timestamps attached to photos shared via *Flickr* to investigate human attention to natural disasters, providing evidence that the number of photos taken and uploaded with a relevant tag, such as “Hurricane Sandy”, may provide an indicator of the severity of the disaster at a given time [16]. A fraction of the photographs uploaded to *Flickr* carry metadata about not only the time, but also the location at which they have been taken. Users who upload photos from a smartphone or from a GPS enabled camera can choose to automatically include the location metadata. Geographic information can also be added after photos have been uploaded on *Flickr*, but this can only be achieved through a manual process, therefore it is likely that the majority of geo-tagged metadata comes from GPS enabled devices. Here, we exploit a dataset totalling more than 140 million pictures uploaded by about 1.7 million *Flickr* users for which both temporal and geographic information is available and quantify the yearly number of visitors to the UK from different countries. This analysis is only one of the many ways in which insights about human mobility can be obtained from the analysis of social media, as the time and location of a large number of individuals can also be employed to discern, for example, the impact of seasonality in travel patterns [8], or the distribution of travel displacements [24].

## Results

Official statistics derived from the International Passenger Survey carried out by the UK Office for National Statistics detail the number of people who visited the UK between 2008 and 2013 along with their country of origin, defined as the country in which a visitor has spent the past year at the time of the survey. To obtain equivalent estimates using data derived from *Flickr*, we consider the sequence of photographs each user has taken and uploaded, and determine where and when each photograph was taken. We use this information to estimate in which country a *Flickr* user spent each day between the date on which the user took their first photograph, and the date on which the user took their final photograph. Where a user took no photographs on a particular day, we assume that the user was still in the country in which they took their previous photograph. For each user, we search for cases in which a photograph was taken in the UK, but the previous photograph was taken in a different country. In such cases, we determine the country in which the user spent the majority of the previous 12 months. We consider this country as the country of origin of the *Flickr* user.

**Fig 1:**
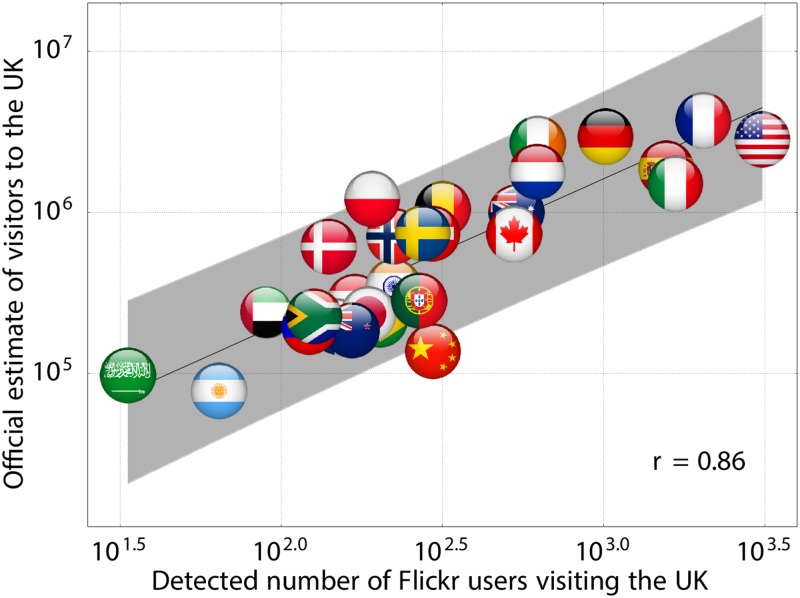
Relationship between official and *Flickr* based estimates of visitors to the UK. We analyse geotagged photos taken and uploaded to *Flickr* between 2008 and 2013. We identify users based in 28 countries outside the UK, and determine the average number of *Flickr* users who visited the UK from each of these countries each year during this period. We find a significant correlation between the detected number of *Flickr* users visiting the UK and the official estimate of visitors to the UK calculated by the Office for National Statistics (*r* = 0.86, *N* = 28, *p* < 0.001, Pearson’s correlation test). The solid line depicts a least-squares fit, and the shaded area represents a 95% confidence interval.

In Fig 1, we depict the average number of *Flickr* users who visited the UK from another country each year between 2008 and 2013, and compare this to the average official estimate of visitors to the UK per year calculated by the Office for National Statistics, for 28 countries for which official estimates are produced (since the distribution of the number of visitors across different countries spans multiple orders of magnitude, we consider logarithmic figures hereafter). We find a significant correlation between the detected number of *Flickr* users visiting the UK and the official estimate of visitors to the UK (*r* = 0.86, *N* = 28, *p* < 0.001, Pearson’s correlation test).

**Fig 2:**
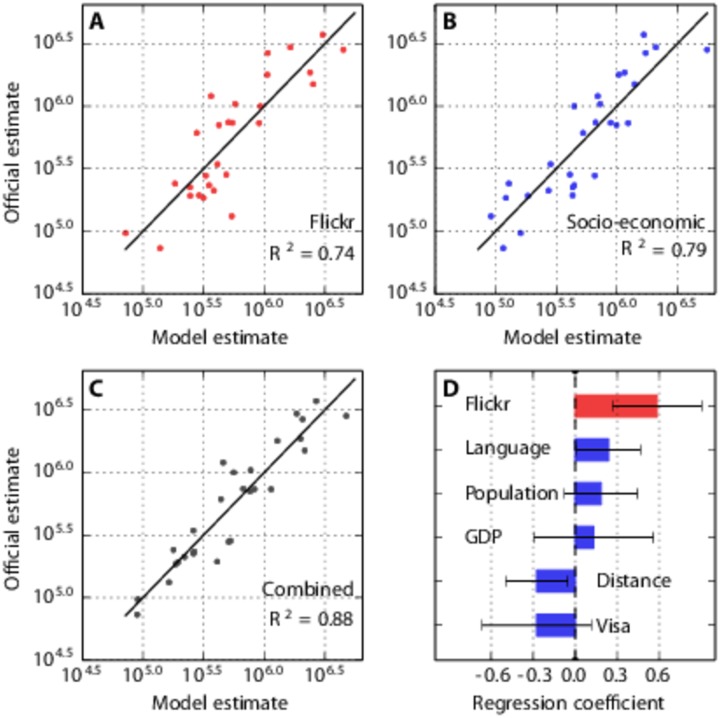
Comparison of estimates of the number of visitors to the UK using standard socio-economic indicators, and estimates using *Flickr* data. We analyse data for all 28 countries of origin depicted in Fig 1, from 2008 to 2013. (**A**) Estimates of the average number of visitors per year to the UK, generated by a regression model using the detected number of *Flickr* users visiting the UK only. (**B**) Estimates generated using five socio-economic indicators, namely whether the country of origin has English as an official language, the population of the country, the GDP per capita of the country, the distance between the largest city in the country and London, and the stringency of UK visa requirements for citizens of the country. (**C**) Estimates generated by a combined model, using both *Flickr* data and the socio-economic indicators. We find that the combined *Flickr* and socio-economic model significantly outperforms both the *Flickr* model (*F*(5,21) = 4.75, *p* < 0.005) and the socio-economic model (*F*(1,21) = 14.57, *p* < 0.005). (**D**) In the combined model, regression coefficients for *Flickr*, language and distance are significantly different from 0. Error bars indicate 95% confidence intervals.

We investigate how estimates of the average number of visitors to the UK per year derived from *Flickr* data compare to estimates of the number of visitors using standard socio-economic indicators. We find no significant difference between the performance of a linear regression model generating estimates from the detected number of *Flickr* users visiting the UK (*R* ^2^ = 0.74, Fig 2A) and the performance of a linear regression model generating estimates using five key socio-economic indicators, namely whether the country of origin has English as an official language, the population of the country, the GDP per capita of the country, the distance between the largest city in the country and London, and the stringency of UK visa requirements for citizens of the country (*R* ^2^ = 0.79, Fig 2B; *F*(4,22) = 1.42, *p* > 0.05). However, we find that estimates of the average number of visitors to the UK per year derived from a combined model using both *Flickr* data and the socio-economic indicators (*R* ^2^ = 0.88, Fig 2C) are significantly more accurate than estimates generated from *Flickr* data (*F*(5,21) = 4.75, *p* < 0.005) or socio-economic data alone (*F*(1,21) = 14.57, *p* < 0.005). We find that information on how far away a country is and whether a country has English as an official language is of particular useful in improving *Flickr*-based estimates (Fig 2D).

We analyse how the relationship between the detected number of *Flickr* users visiting the UK and the official estimate of visitors to the UK changes from year to year. We find that while there are significant differences in the number of overall visitors in different years (*F*(5,161) = 4.59, *p* < 0.001, one way ANOVA), the parameters of a linear regression model using *Flickr* data to estimate the number of visitors from each country are relatively stable from year to year (S1 Fig). However, the change in the official estimate of the number of visitors to the UK from each individual country each year is relatively small (S2 Fig). We find no evidence that a model using data from *Flickr* is able to capture these differences (slope *β* = −0.04, *t* = −0.99, *p* > 0.05, linear regression model with different intercepts for each country). While this finding may indicate limitations of the power of *Flickr* data to provide estimates at this level of precision, we note that the average number of *Flickr* users we analyse each year (15,244) is of the same order of magnitude as the average yearly number of Office for National Statistics International Passenger Survey participants (40,259). The possibility therefore exists that noise at this level of precision could be originating from either the *Flickr* derived or the official estimates. The change in number of visitors to the UK across different countries of origin is in general much larger than the variation across different years for the same country, at least for the period considered in this study. We argue, therefore, that it is in general more important to estimate differences across countries than to track slowly-changing yearly figures for individual countries. There can be situations, however, where policy makers might specifically seek to understand dynamics relative to individual countries, for example when evaluating the impact of visa policy changes. In this case, increased investment might be required to collect data from larger surveys or, based on the success of this study, additional online sources such as *Twitter* or *FourSquare* may be explored to extend the method presented in this work.

## Conclusions

Data on usage of online services offer measurements of human behaviour at near to no cost with minimal delay. In contrast, many important governmental statistics are currently generated using expensive and time consuming surveys. We investigate whether data from geo-tagged photographs uploaded to the photo sharing website *Flickr* could be used to estimate the number of visitors to the UK from different countries. Our results provide evidence of a correlation between the detected number of *Flickr* users visiting the UK from different countries and official visitor estimates calculated by the UK Office for National Statistics, when considering data aggregated across the years 2008 to 2013. We further find that data on the time and location at which *Flickr* photographs are taken can be used to generate more accurate estimates of the average number of visitors to the UK per year than estimates generated from standard socio-economic indicators alone. However, our analyses also show that the official survey based estimates of the small yearly changes in the number of visitors to the UK from individual countries do not correspond to the small yearly changes found in the *Flickr* data. With this caveat, our findings underline the potential for indicators of key aspects of human behaviour, such as mobility, to be generated from data attached to the vast volumes of photographs posted online.

## Supporting Information

S1 Fig Relationship between official and *Flickr* based estimates of visitors to the UK by year. We analyse geotagged photos taken and uploaded to *Flickr* between 2008 and 2013. We identify users based in 28 countries outside the UK, and determine the number of *Flickr* users who visited the UK from each of these countries each year during this period. We find a significant correlation between the detected number of *Flickr* users visiting the UK and the official estimate of visitors to the UK calculated by the Office for National Statistics for each year. We further note that the parameters of the models remain relatively stable between years. The solid lines depict least-squares fits, and the shaded areas represent 95% confidence intervals.
(PDF)

S2 Fig Relationship between official and *Flickr* based estimates of visitors to the UK by country. For each of the 28 countries analysed, we depict the relationship between the yearly detected number of *Flickr* users visiting the UK and the yearly official estimate of visitors to the UK calculated by the Office for National Statistics. We plot one data point for each of the six years between 2008 and 2013. The country labels follow the ISO3166-1 alpha-3 standard, and all the axis limits are set to span the whole range of values in the data, so that the position of different countries in Fig 1 of the main manuscript can be inferred by the position of the points in each subplot. We note that within countries, the change in the number of visitors from year to year is relatively small. We find no evidence that yearly changes in the *Flickr* estimate of the number of visitors correlate with yearly changes in the official estimates of the number of visitors. However, we observe that the average number of *Flickr* users we analyse each year (15,244) is of the same order of magnitude as the average yearly number of Office for National Statistics International Passenger Survey participants (40,259). The possibility therefore exists that noise at this level of precision could be originating from either the *Flickr* derived or the official estimates.
(PDF)

S3 Fig Time window analysis. we have analysed the trajectories of *Flickr* users by using a mode filter window that takes into account a time interval and returns the most common geographic location within the interval. For each window length in days, the resulting number of relocations per year represents the number of times two different countries appear in the filtered trajectory. Therefore, considering a window length of one year that we used in our method, the median number of relocations is around 0.3, and the 75th percentile of users made less than 0.5 relocations per year.
(PDF)

S1 Dataset Flickr and official estimates of the number of visitors to the UK. This file contains the official estimates of the number of visitors to the UK, the number of *Flickr* users who visited the UK from different countries, and the socio-economic indicators discussed in the main text.
(CSV)

S1 Text Description of materials and methods. (PDF)
