# Supplementary figures and images for: Quantifying International Travel Flows Using Flickr
Source: PLoS One. 2015 Jul 6;10(7):e0128470. doi: 10.1371/journal.pone.0128470 (PMC4493158; doi:10.1371/journal.pone.0128470)

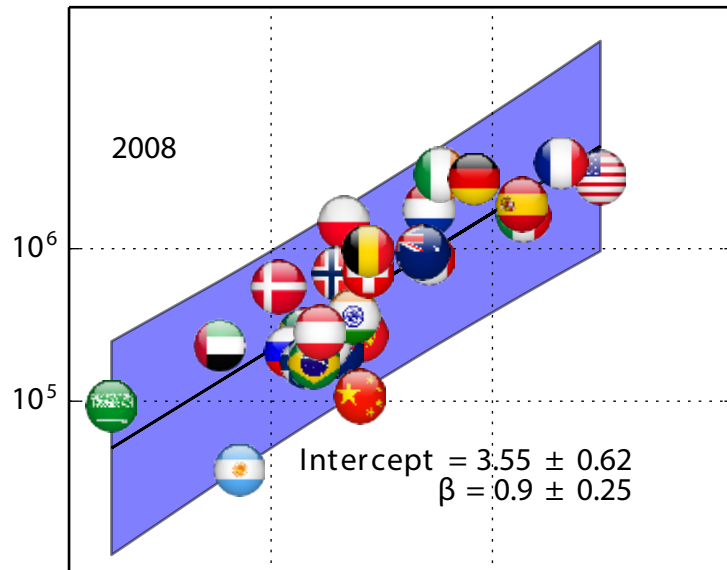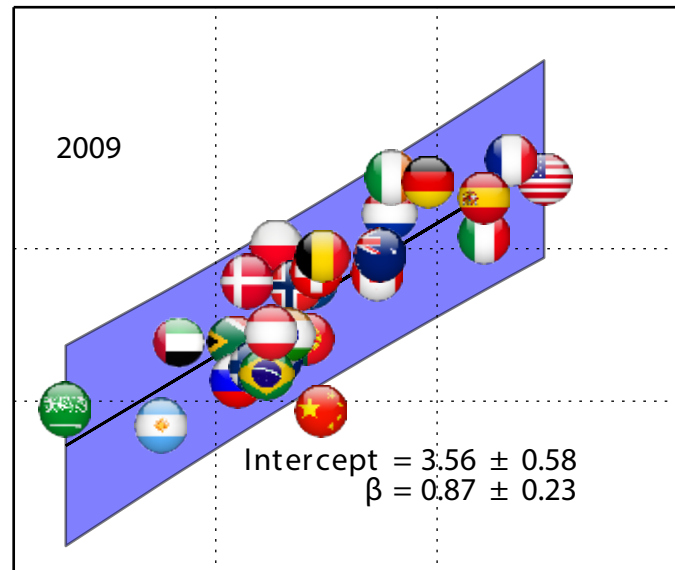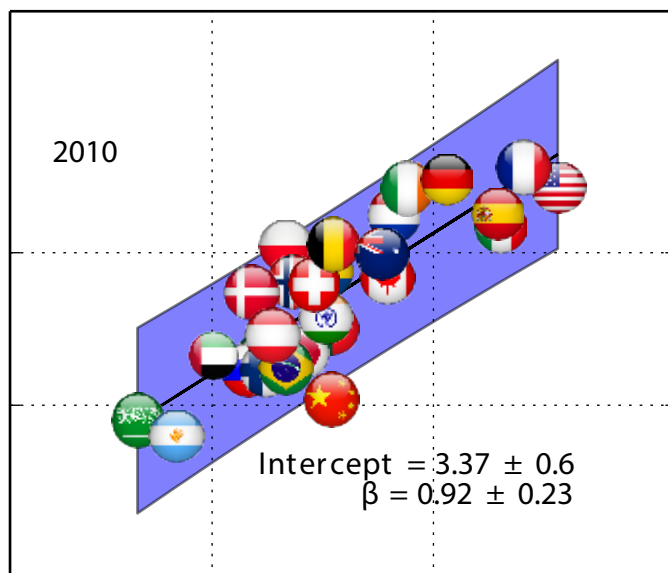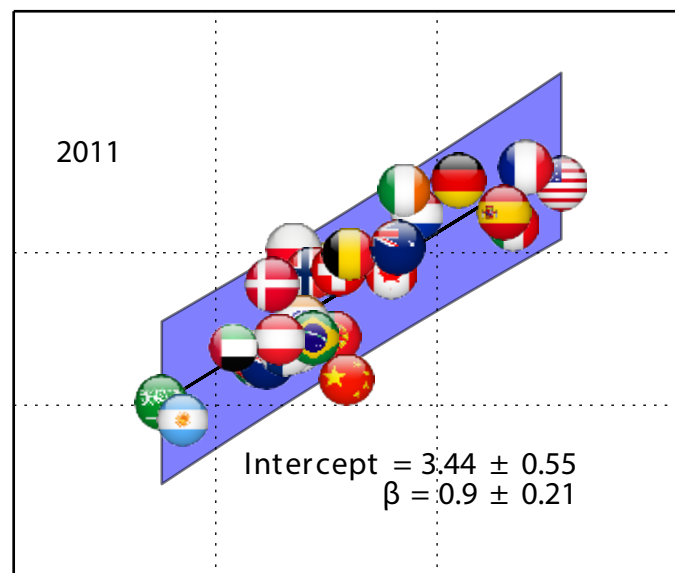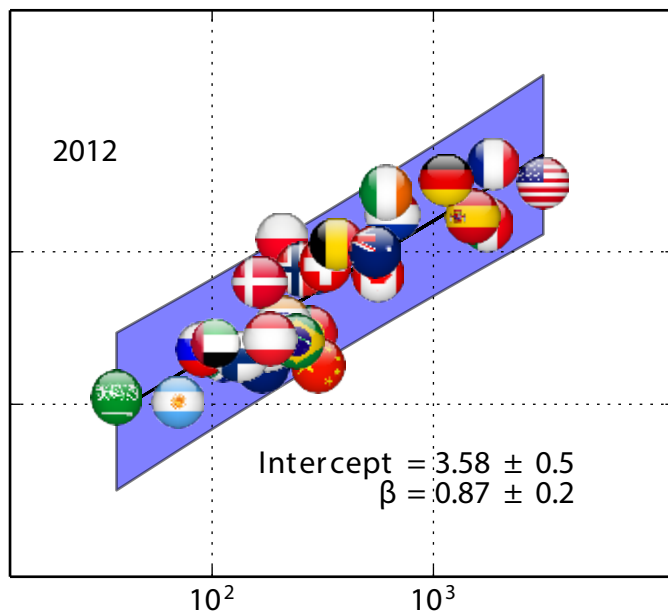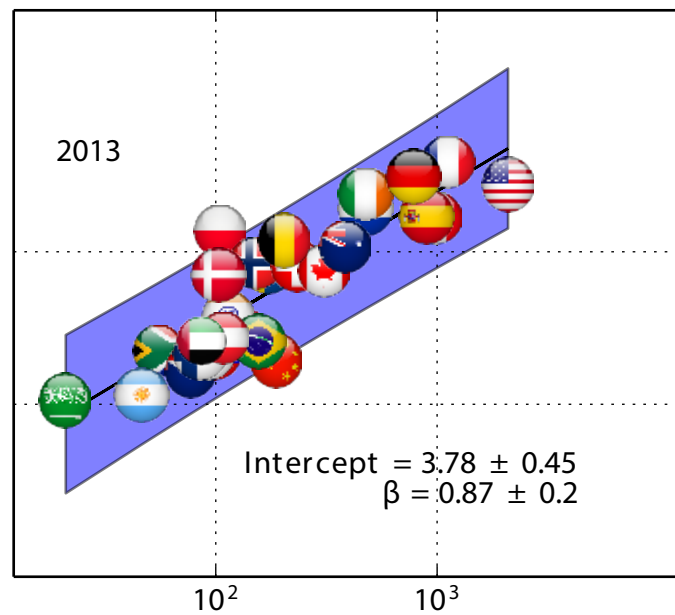

Detected number of Flickr users visiting the UK

Supplement: S1 Fig — We analyse geotagged photos taken and uploaded to Flickr between 2008 and 2013. We identify users based in 28 countries outside the UK, and determine the number of Flickr users who visited the UK from each of these countries each year during this period. We find a significant correlation between the detected number of Flickr users visiting the UK and the official estimate of visitors to the UK calculated by the Office for National Statistics for each year. We further note that the parameters of the models remain relatively stable between years. The solid lines depict least-squares fits, and the shaded areas represent 95% confidence intervals. (PDF) [file pone.0128470.s001.pdf]

Official estimate of visitors to the UK

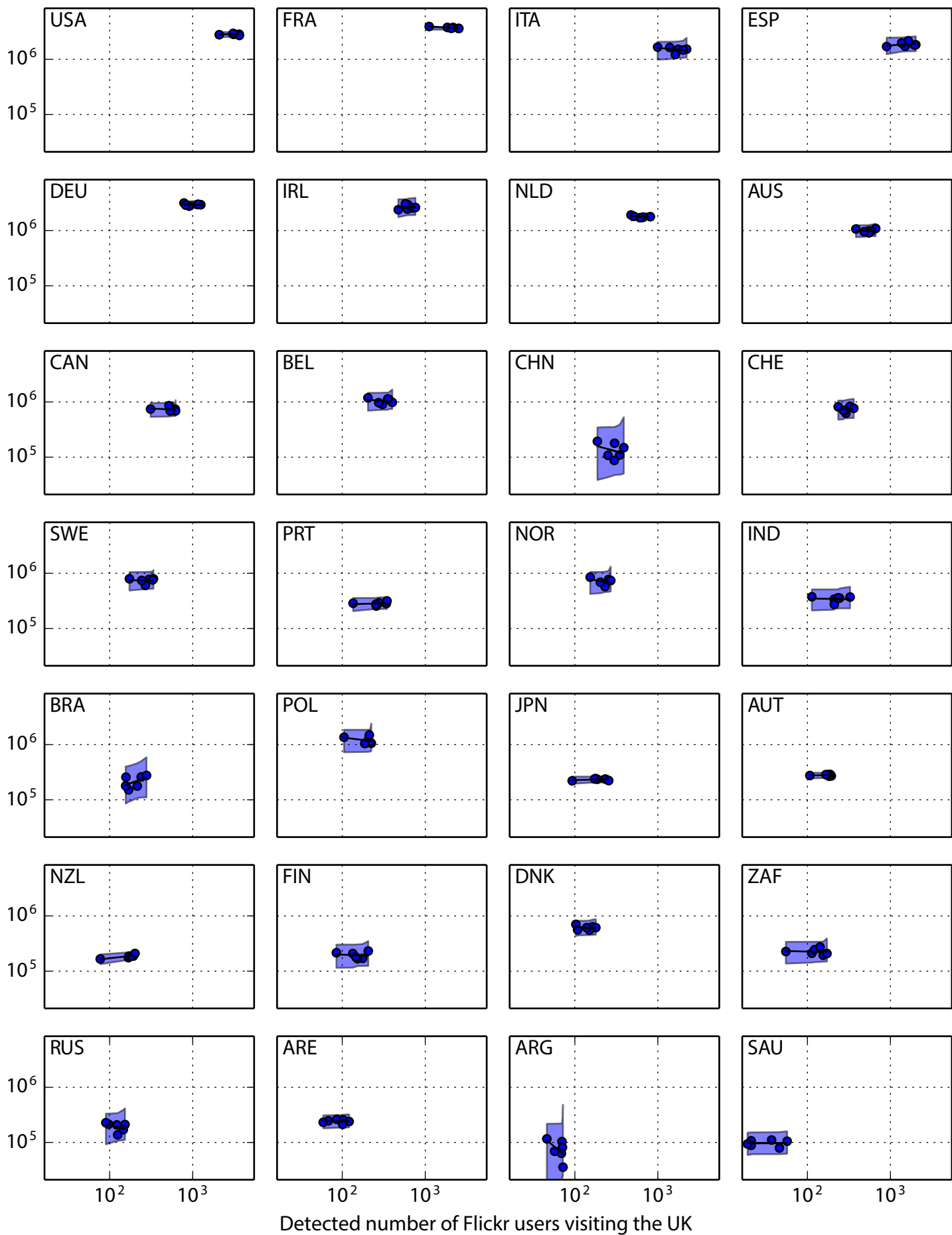

Supplement: S2 Fig — For each of the 28 countries analysed, we depict the relationship between the yearly detected number of Flickr users visiting the UK and the yearly official estimate of visitors to the UK calculated by the Office for National Statistics. We plot one data point for each of the six years between 2008 and 2013. The country labels follow the ISO3166-1 alpha-3 standard, and all the axis limits are set to span the whole range of values in the data, so that the position of different countries in Fig 1 of the main manuscript can be inferred by the position of the points in each subplot. We note that within countries, the change in the number of visitors from year to year is relatively small. We find no evidence that yearly changes in the Flickr estimate of the number of visitors correlate with yearly changes in the official estimates of the number of visitors. However, we observe that the average number of Flickr users we analyse each year (15,244) is of the same order of magnitude as the average yearly number of Office for National Statistics International Passenger Survey participants (40,259). The possibility therefore exists that noise at this level of precision could be originating from either the Flickr derived or the official estimates. (PDF) [file pone.0128470.s002.pdf]

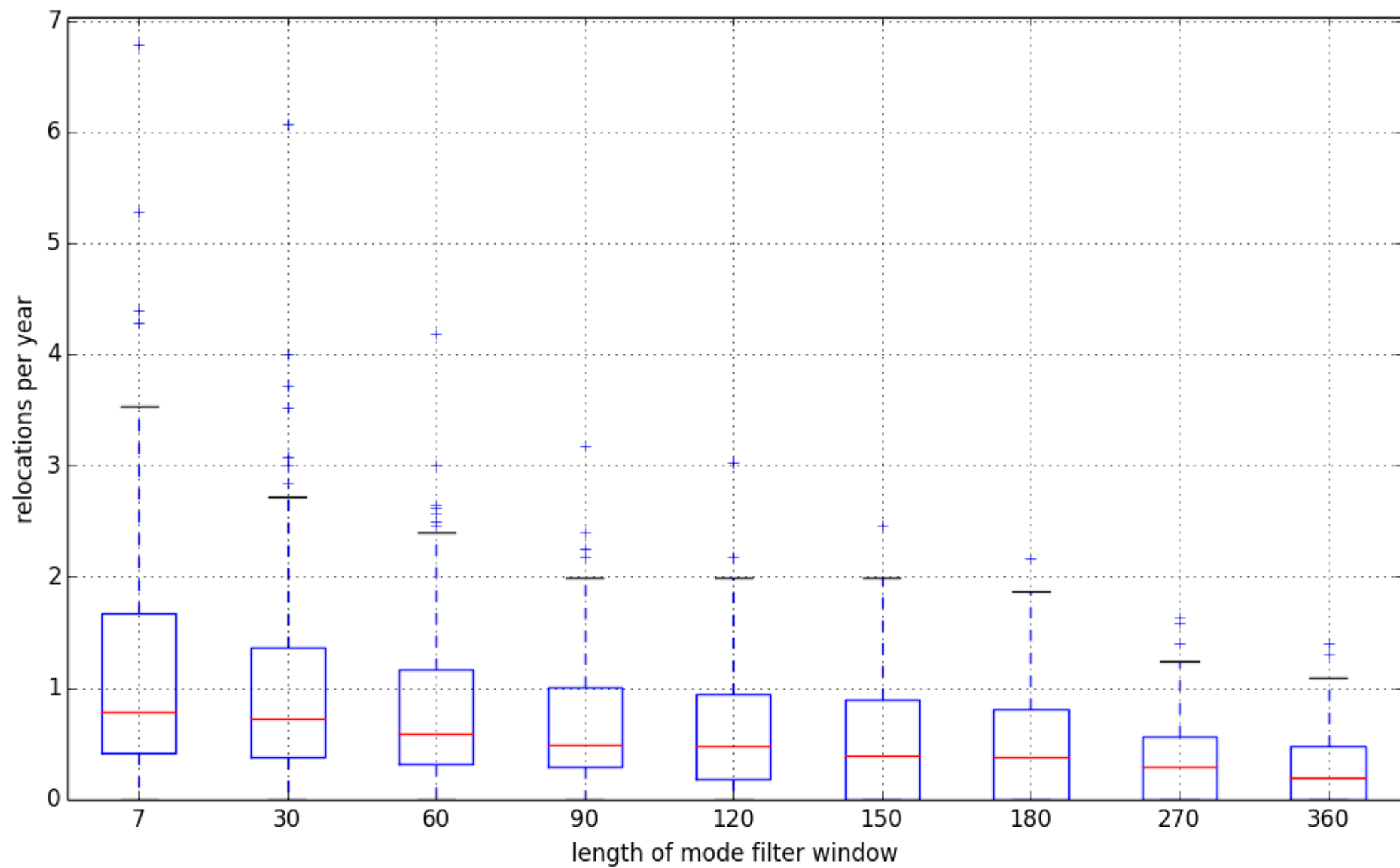

Supplement: S3 Fig — we have analysed the trajectories of Flickr users by using a mode filter window that takes into account a time interval and returns the most common geographic location within the interval. For each window length in days, the resulting number of relocations per year represents the number of times two different countries appear in the filtered trajectory. Therefore, considering a window length of one year that we used in our method, the median number of relocations is around 0.3, and the 75th percentile of users made less than 0.5 relocations per year. (PDF) [file pone.0128470.s003.pdf]
