# Supplementary material for: Quantifying International Travel Flows Using Flickr
Source: PLoS One. 2015 Jul 6;10(7):e0128470. doi: 10.1371/journal.pone.0128470 (PMC4493158; doi:10.1371/journal.pone.0128470)
Supplement: S1 Text — (PDF) [file pone.0128470.s005.pdf]

Quantifying International Travel Flows Using Flickr (Supplementary Material)

Daniele Barchiesi[1,*], Helen Susannah Moat[2], Christian Alis[1], Steven R. Bishop[1] and Tobias Preis[2]

1 Department of Mathematics, University College London, London, United Kingdom

2 Warwick Business School, University of Warwick, Coventry, United Kingdom

∗ E-mail: d.barchiesi@ucl.ac.uk

Materials and Methods

Retrieval of ***Flickr*** data.

We used the `flickr.photo.search` API to download metadata on all the publicly viewable photographs uploaded on the site between 2007 and 2013, for which both timestamps and geographic coordinates were available. The dataset was downloaded in March 2014. The resulting dataset consists of `json` records describing more than 140 million pictures uploaded by about 1.7 million users.

To compute the country in which each photograph was taken, we used country boundaries downloaded from Natural Earth Data in April 2014 [1].

Some photographs had coordinates which were associated to a sea location rather than a land location. These photographs were not associated to a country, and were therefore removed from our analysis. We also removed users who only uploaded photographs on a single day. The data remaining for analysis describes photographs taken by roughly 1 million users. To quantify the number of people who visited the UK, each year we selected only the users who at some point during the year uploaded a photo taken in the UK. The average number of users per year is 15244.

Retrieval of official travel data

The official number of visitors by country of origin was obtained from the *VisitBritain* website [2]. All the countries of origin reported in these statistics were considered except from Honk Kong and Singapore because their limited geographic area corresponded with significant omissions when converting geographic coordinates to their country.

Retrieval of socio-economic data

Geographic, social and economic indicators were obtained from the following sources in June 2014.

Population and *GDP* per capita at purchasing power parity Data on population and GDP were obtained from the World Bank [3] using the Python `pandas.io` library. The value of *GDP* for Argentina was obtained from Wikipedia [4] because unavailable from the World Bank figures.

Language The list of countries where English is an official language was retrieved from the *Wikipedia* article "List of countries where English is an official language" [5].

Distance The name of the largest city for each country was obtained from each country's page on *Wikipedia*. The distance from each city to London was obtained from *DistanceFromTo* [6].

Visa The stringency of visa regulations was mapped to a scale from 0 (less stringent) to 3 (more stringent). The definitions of these levels were as follows:

0 : Freedom of movement for workers

1 : Visa not required for touristic visits

2 : Visa required for visits

3 : Visa required for transit and visits

A list of countries with freedom of movement for workers coming to the UK was obtained from *Wikipedia* [7]. Other visa requirements were obtained from the UK Home Office [8].

References

1. http://www.naturalearthdata.com/downloads/110m-cultural-vectors/.
2. `http://www.visitbritain.org/bounce.aspx?PG=/Images/region%20England%202013_tcm29-14618.xlsx`.
3. http://data.worldbank.org/.
4. `http://en.wikipedia.org/wiki/List_of_countries_by_GDP_(PPP)_per_capita`.
5. http://en.wikipedia.org/wiki/List_of_countries_where_English_is_an_official_language.

6. http://www.distancefromto.net/.

7. http://en.wikipedia.org/wiki/Freedom_of_movement_for_workers.

8. https://www.gov.uk/government/organisations/uk-visas-and-immigration.

1 Figures

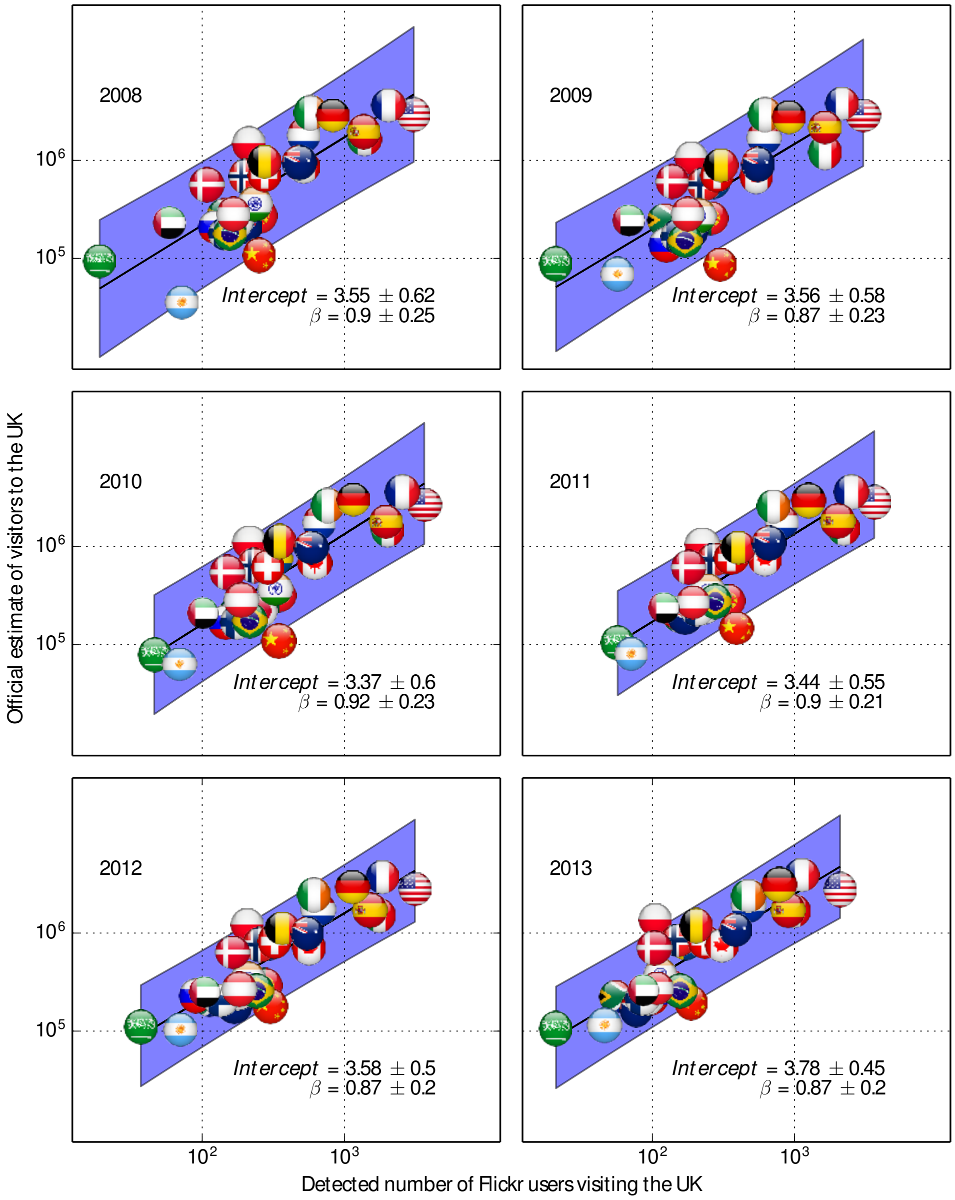

Figure 1. Relationship between official and *Flickr* based estimates of visitors to the UK by year. We analyse geotagged photos taken and uploaded to *Flickr* between 2008 and 2013. We identify users based in 28 countries outside the UK, and determine the number of *Flickr* users who visited the UK from each of these countries each year during this period. We find a significant correlation between the detected number of *Flickr* users visiting the UK and the official estimate of visitors to the UK calculated by the Office for National Statistics for each year. We further note that the parameters of the models remain relatively stable between years. The solid lines depict least-squares fits, and the shaded areas represent 95% confidence intervals.

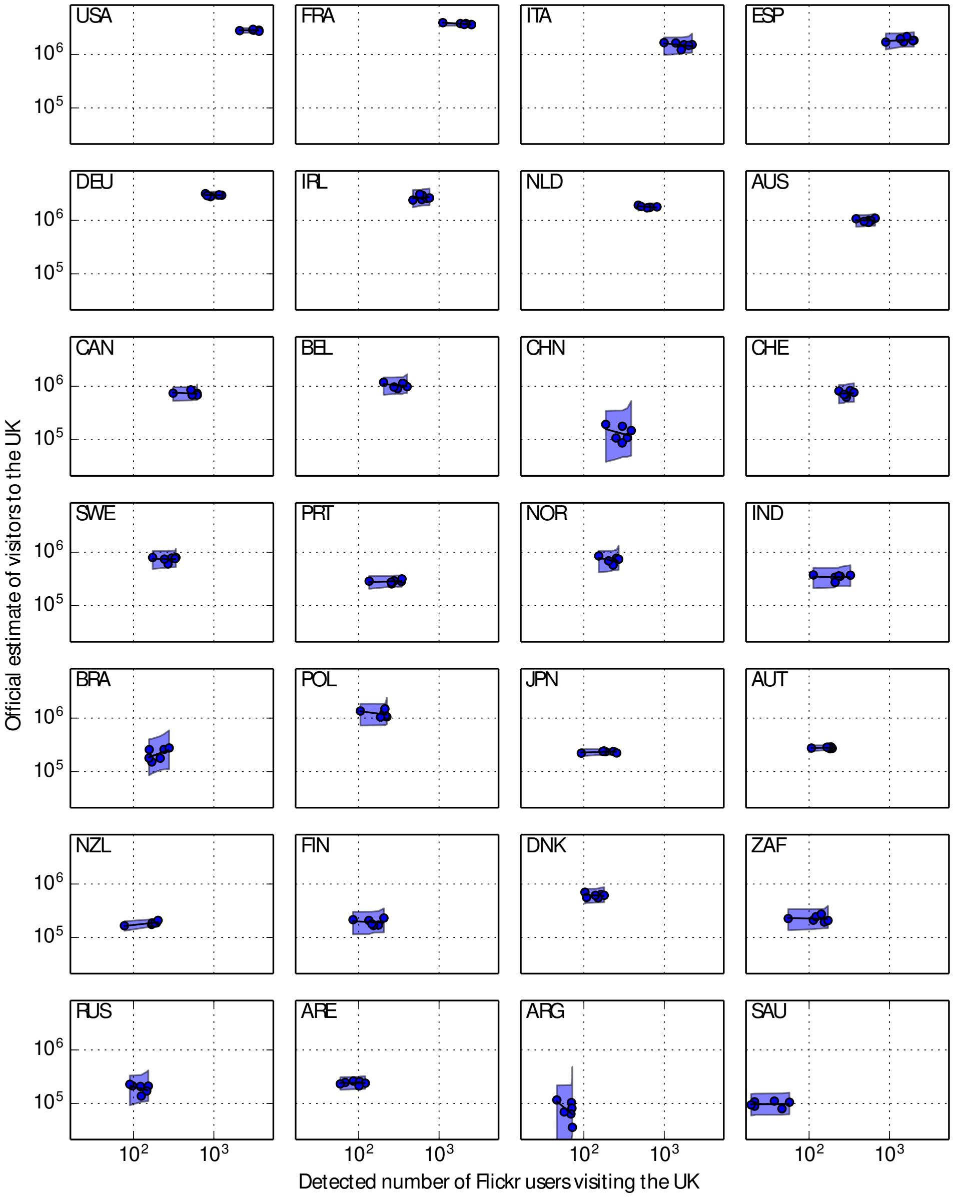

Figure 2. Relationship between official and *Flickr* based estimates of visitors to the UK by country. For each of the 28 countries analysed, we depict the relationship between the yearly detected number of *Flickr* users visiting the UK and the yearly official estimate of visitors to the UK calculated by the Office for National Statistics. We plot one data point for each of the six years between 2008 and 2013. The country labels follow the ISO3166-1 alpha-3 standard, and all the axis limits are set to span the whole range of values in the data, so that the position of different countries in Fig. 1 of the main manuscript can be inferred by the position of the points in each subplot. We note that within countries, the change in the number of visitors from year to year is relatively small. We find no evidence that yearly changes in the *Flickr* estimate of the number of visitors correlate with yearly changes in the official estimates of the number of visitors. However, we observe that the average number of *Flickr* users we analyse each year $(15, 244)$ is of the same order of magnitude as the average yearly number of Office for National Statistics International Passenger Survey participants $(40, 259)$. The possibility therefore exists that noise at this level of precision could be originating from either the *Flickr* derived or the official estimates.
